# Longitudinal Impact of Adjuvant Radiation Therapy on Financial Toxicity in African-American Breast Cancer Patients: Early Findings From the Navigator-Assisted Hypofractionation (NAVAH) Program Phase I Clinical Trial

**DOI:** 10.7759/cureus.94976

**Published:** 2025-10-20

**Authors:** Maya J Stephens, Ursula J Burnette, Nimisha Kasliwal, Louisa Onyewadume, Shearwood McClelland

**Affiliations:** 1 Medical Student, Augusta University Medical College of Georgia, Augusta, USA; 2 Department of Radiation Oncology, University of Oklahoma College of Medicine, Oklahoma City, USA; 3 Department of Radiation Oncology, Case Western Reserve University School of Medicine, Cleveland, USA; 4 Department of Radiation Oncology, Duke University Medical Center, Durham, USA; 5 Department of Neurological Surgery, University of Oklahoma College of Medicine, Oklahoma City, USA

**Keywords:** breast cancer, financial toxicity, general radiation oncology, patient navigation, radiation therapy

## Abstract

Purpose/objectives

Breast cancer remains the most prevalent cancer among women, with African Americans experiencing the highest rates of mortality/morbidity. In this context, our current study examines financial toxicity (FT) among African American breast cancer patients before and one month after undergoing adjuvant radiation therapy (RT), aiming to assess the impact of RT on quality of life. This IRB-approved cohort study is part of an ongoing phase I clinical trial evaluating the role of patient navigation during RT as part of the Navigator-Assisted Hypofractionation (NAVAH) program.

Methods

Adults of African American race with a pathologically confirmed diagnosis of early-stage breast cancer following lumpectomy were recruited for enrollment before receipt of adjuvant RT. As part of the NAVAH clinical trial, each participant was paired with a trained patient navigator for support throughout and following RT. FT was assessed using the 12-item COmprehensive Score for financial Toxicity-Functional Assessment of Chronic Illness Therapy (COST-FACIT) questionnaire. This validated instrument was administered (in person or via telephone) at two time points: prior to RT initiation and at one month following completion. Composite COST-FACIT scores were calculated. Paired comparisons of pre- and post-treatment scores were conducted to evaluate FT changes over time.

Results

A total of 40/40 (100%) participants completed the pre-RT COST-FACIT assessment (score range: 4.4-39). At one-month follow-up, post-treatment data was available for 26/40 (65%) participants (score range: 6-38). Prior to starting RT, 24/40 (60%) of participants reported experiencing FT, compared to 16/26 (62%) following treatment (p<0.05). Among those who completed both time points, 9/26 (35%) demonstrated an improvement in FT scores, while 3/26 (11.5%) reported none. However, 14/26 (54%) of participants experienced FT worsening. Comparison revealed no significant difference in FT for one versus three or more weeks of RT at one-month post-treatment (p>0.05).

Conclusion

These findings from the first study longitudinally assessing FT in breast cancer patients receiving RT highlight the dynamic nature of FT among this patient population. While a subset of patients experienced improvements in financial well-being following treatment, more than half reported worsening financial strain. Noteworthy is that there was no significant difference in FT onset following RT completion. These results emphasize the persistent and evolving burden of financial toxicity for people with cancer, especially this population, reinforcing the need for early, sustained, and culturally responsive interventions. Continued investigation through the NAVAH program will further elucidate the role of patient navigation in mitigating financial hardship and optimizing cancer care delivery for all patients.

## Introduction

Breast cancer is the most frequently diagnosed cancer among women worldwide and remains a leading cause of cancer-related death. In the United States, significant disparities persist in breast cancer outcomes, with African Americans experiencing the highest rates of mortality and delayed diagnosis/treatment [1]. Socioeconomic barriers, including limited access to care, insurance status, social support, provider-based challenges, errant provider impressions of patient cancer screening knowledge [2], impaired patient-physician trust [3], and financial stress [4], contribute to these disparities and hinder optimal outcomes.

One emerging concern related to the economic burden of cancer is financial toxicity (FT). FT refers to the distress or hardship patients experience due to cancer care costs, including direct medical expenses and indirect costs such as lost income, transportation, and caretaker responsibility [5]. Studies have shown that FT adversely impacts quality of life, adherence to treatment, and overall survival [6-7]. Despite growing awareness, FT remains under-researched.

Radiation therapy (RT) is a critical component of breast cancer treatment for patients who desire to keep their affected breast. As part of breast conservation therapy, RT is typically administered following lumpectomy to optimally reduce the risk of recurrence, as RT + lumpectomy has been demonstrated as equivalent to mastectomy [8,9]. However, the treatment often requires daily visits over several weeks, posing significant logistical and financial challenges, especially for individuals facing economic constraints [10]. To our knowledge, no prior studies have specifically evaluated FT before versus after RT in breast cancer patients.

To address this gap, we conducted a prospective study as part of the ongoing Navigator-Assisted Hypofractionation (NAVAH) clinical trial. NAVAH is a phase I trial designed to evaluate the impact of patient navigation on treatment experiences and outcomes for African American breast cancer patients undergoing RT. Previous work from the NAVAH trial has documented evidence of baseline patient FT prior to receipt of RT, including the impact of chemotherapy on FT and comparison of personal versus familial FT [11-12]. Here, we present our initial findings on FT at two time points: prior to RT and one month following completion.

## Materials and methods

Study design and participants

This prospective IRB-approved cohort study was conducted as part of the NAVAH phase I clinical trial (clinicaltrials.gov number NCT05978232) as previously described [1]. Eligible participants were African American adults (age 18+ years) who had a pathologically confirmed diagnosis of early-stage breast cancer. All were scheduled to receive adjuvant RT following lumpectomy. Participants were recruited in person in the oncology setting in a large academic healthcare system. 

Intervention

As part of NAVAH, each enrolled participant was paired with a patient navigator. Navigators provided logistical assistance, emotional support, and resource connection throughout RT, including one month post-treatment and beyond.

Measure

Financial toxicity was measured using the COmprehensive Score for financial Toxicity-Functional Assessment of Chronic Illness Therapy (COST-FACIT) tool, a validated 12-item questionnaire [12]. Higher scores reflect better financial well-being and lower levels of toxicity. Participants completed the survey at two points: prior to initiation of RT and one month following treatment completion. Surveys were administered in person or by telephone, depending on patient availability.

Statistical analysis

Descriptive statistics summarized participant characteristics and COST-FACIT scores. Paired t-tests were used to compare pre-and post-treatment FT scores. Changes in FT were categorized as improved, unchanged, or worsened. P-values less than 0.05 were considered statistically significant.

## Results

Participant characteristics

A total of 40/40 (100%) participants completed the pre-treatment COST-FACIT survey, with FT scores ranging from 4.4 to 39. Of these, 26/40 (65%) participants completed the one-month post-treatment assessment (score range: six -38). The remaining 14/40 (35%) participants were lost to follow-up. Data is listed in Table 1. This table summarizes the number and percentage of participants who completed the COST-FACIT survey at baseline (Pre-RT) and at one-month follow-up (Post RT).

**Table 1:** Participant Completion of COST-FACIT RT: radiation therapy; COST-FACIT: COmprehensive Score for financial Toxicity-Functional Assessment of Chronic Illness Therapy

	Number of Participants N (%)
Pre-RT	40 (100%)
One-month Post-RT	26 (65%)
Unavailable at 1-month Post-RT	14 (35%)
Total	40 (100%)

Financial toxicity before and after RT

At baseline, 24/40 (60%) of participants reported experiencing some degree of financial toxicity. One month after completing RT, 16/26 (62%) of participants reported FT, representing a statistically significant change (p<0.05). Among participants with paired pre- and post-treatment data, 9/26 (35%) reported an improvement in FT scores while 3/26 (12%) experienced no change. In total, 14/26 (54%) reported worsening FT. Overall, the distribution of FT changes indicates considerable variability in patient experiences. Comparison revealed no significant difference in FT for one versus three or more weeks of RT at one month post-treatment (p>0.05). Data is listed in Table 2.

**Table 2:** Financial toxicity (FT) Among Patients Before and After Radiation Therapy (RT)

Measure	Number of Participants N (%)
FT at Baseline	24 (60%)
FT at One-Month Follow-Up	16 (62%)
Participants with Paired Treatment Data	26 (65%)
Participants with Improved FT	9 (35%)
Participants with No Change in FT	3 (12%)
Participants with Worsened FT	14 (54%)

## Discussion

This study represents the first investigation of financial toxicity over time in breast cancer patients receiving RT. Our findings reveal that FT remains a prevalent and evolving burden, with more than half of participants experiencing a decrease in financial well-being upon completing treatment. This highlights the need to continue to explore FT and its impact on patients with breast cancer. While 9/26 (35%) patients reported improved FT, a larger proportion [14/26 (54%)] experienced an increase in their cancer-related financial burden. This may reflect accumulating indirect costs (e.g., lost wages, transportation) or delays in accessing financial support resources. Although RT did not increase the incidence of FT, these results emphasize that RT may pose additional economic strain to patients already exhibiting FT at baseline, particularly for populations already experiencing inequities. Our findings may indicate that RT has less impact than chemotherapy on FT for this patient population, given our previous work, which highlighted FT amongst patients undergoing RT [11], although RT and chemotherapy have yet to be compared directly to answer this question.

Patient navigation, a core component of the NAVAH program, may offer a promising approach to mitigate FT. While this study did not evaluate navigator-specific effects on FT, the ongoing trial will investigate how having access to a patient navigator correlates with financial burdens. Our study has several strengths, including its prospective design, use of a validated FT measure, and focus on a high-risk population.

Limitations

Limitations of this study include a small sample size, data derived from a single institution, impaired generalizability of our findings to patients of all races, a focus on breast cancer patients, potentially limiting generalizability to other cancer disease sites, and the number of participants lost to follow-up. An additional limitation involved the recent transition of the NAVAH trial from University Hospitals to the University of Oklahoma, resulting in limited access to longitudinal data from some enrolled patients; we anticipate that this contributed to the unexpectedly high attrition rate reported in this analysis. We expect future findings to reflect improved longitudinal attrition within the University of Oklahoma cohort.

## Conclusions

This is the first longitudinal study to assess FT in breast cancer patients undergoing RT. While some patients with FT prior to RT experienced improvements, the majority reported worsening FT, highlighting the complex socioeconomic challenges faced throughout treatment; however, patients without FT prior to RT were not significantly more likely to develop FT following RT. These findings emphasize the importance of early, continuous, and culturally aware interventions to address FT while supporting RT as having relatively minimal impact on FT in the short-term period following treatment.

The NAVAH program represents a critical step toward reducing disparities in cancer care through patient navigation. Continued investigation will explore the impact of navigation on FT and expand to include additional high-risk populations and other cancer types. By beginning with the highest-risk population for breast cancer mortality, our ultimate goal is to dismantle financial barriers to cancer survival and ensure optimal guideline-concordant care for all.
